# Materials count: Linear-spatial materials improve young children’s addition strategies and accuracy, irregular arrays don’t

**DOI:** 10.1371/journal.pone.0208832

**Published:** 2018-12-31

**Authors:** Joanna Schiffman, Elida V. Laski

**Affiliations:** Department of Counseling, Developmental, and Educational Psychology, Boston College, Chestnut Hill, Massachusetts, United States of America; University of New England, AUSTRALIA

## Abstract

Children who use advanced arithmetic strategies, such as count-on and decomposition, are more accurate when solving arithmetic problems and are more likely to later have higher levels of math achievement. The present study tested the hypothesis that instruction using linear-spatial representations would activate children’s knowledge necessary for use of mental addition strategies and, thus, lead to greater accuracy on addition problems, than instruction using irregular representations of magnitude. As predicted, low-income kindergartners (*n* = 29) randomly assigned to practice sums up to 10 using materials that instantiated the linear-spatial features of a mental number line (i.e., discrete squares arranged in rows) demonstrated substantially more improvement in solving unpracticed addition problems than children who practiced with irregular materials (i.e., pictures of stars arranged in random arrays). This was particularly true for children with better initial numerical knowledge, which provided support for the idea that existing knowledge was activated. The use of count-on more than doubled from pretest to posttest among children in the linear-spatial condition and this mediated the difference in improvement between conditions. The importance of aligning instructional materials to relevant mental representations–consistent with the Cognitive Alignment Framework for instructional design—is discussed.

## Introduction

Only one third of American eighth-graders are proficient in mathematics [1]. This poor performance has its roots in early chilhood; kindergartners’ mathematical knowledge, and arithmetic knowledge specifically, predicts subsequent mathematics achievement test scores through high school [2–6]. Thus, identifying effective approaches for improving young children’s arithmetic knowledge could have lasting effects on mathematics success. Further, it could be particularly true for children from lower-income families who solve symbolic addition problems substantially less accurately and who use ineffective addition strategies far more frequently than more affluent children [7–10].

The goal of the present study was to develop and test an instructional intervention for arithmetic, using the cognitive alignment approach. The cognitive alignment approach provides a theoretical framework for considering which instructional materials are most likely to produce effective learning [11]. Its basic principle is the more precisely physical materials and learning activities align with the desired mental representation, the more likely students are to acquire that representation. Support for the premise of cognitive alignment comes from research on analogical reasoning [12,13]. For example, physical characteristics and instructions that prompt children to notice the alignment of key relations in one problem with key relations in another increase children’s identification of analogous solution procedures on yet other, parallel problems [12,14]. Thus, the logic is that greater alignment in the features of instruction and mental representations facilitates the mapping between them and promotes learning.

In many learning situations, however, the desired outcome is not a mental representation but rather a procedure or strategy. In the case of arithmetic, a key learning goal is the ability to execute more advanced problem-solving strategies in order to improve accuracy and efficiency. An understanding of numerical magnitude is assumed to be an underlying mental representation that contributes to arithmetic accuracy [15,16], but in arithmetic instruction is not a goal in and of itself. In accordance with the cognitive alignment framework, we hypothesized that arithmetic instruction that instantiates the key properties of mental representations of numerical magnitude (e.g., linearly increasing, spatially organized from left to right)–reflecting alignment to the desired mental representation–should be more likely to facilitate the use of better arithmetic strategies, and thus improve accuracy, than instruction that does not. Testing this hypothesis provided an opportunity to explore whether the cognitive alignment approach extends beyond situations in which there is a direct mapping between the physical materials and the desired mental representation (e.g., linear number line game to linear number line representations) [11,17] to those where there is an indirect mapping via an intermediary representation underlying a desired procedural outcome (e.g., linear-spatial materials to linear magnitude representations to arithmetic strategies).

### Addition strategies

A good deal of mathematics instruction in kindergarten and first grade is dedicated to practice with addition and to helping children acquire addition strategies [18]. There are four main types of addition strategies: count-all, count-on, decomposition, and retrieval [19–21]. The *count-all* strategy involves counting out each addend and then counting the total (e.g., to solve 4+3 a child would first count to 4, then count to 3, and then count from 1 to 7). The *count-on* strategy involves counting up from one addend the value of the second addend (e.g., to solve 4+3 a child would count 5, 6, 7). *Decomposition* involves transforming the original problem into two or more simpler problems, using either a previously memorized number fact or the base-10 properties of the number system (e.g., to solve 4+7 a child might first add 4+6 to get 10 and then add 1 more). The last strategy, *retrieval*, involves recalling the solution from memory.

While children can use multiple strategies at any given time, there is a general progression during the first years of elementary school from using basic strategies toward using more advanced ones [22–24]. Initially, children solve most addition problems using the count-all strategy. They then tend to move toward using count-on as the predominant strategy. Later, retrieval and decomposition become most prevalent [24]. *Count-on*, *decomposition*, and *retrieval* constitute more-advanced strategies because they all benefit from the mental representation of at least one of the addends. For example, to solve 8 + 4 using count-on, 8 can be represented mentally and then 4 counted-on to arrive at 12. Although some children rely on concrete representation of one addend (e.g., holding up 8 fingers before counting on 4), children who rely on mental representations of magnitude should exhibit greater flexibility when using count-on and be better able to transition to other mental strategies. Likewise, decomposition (e.g., solving 8+ 4 by representing 4 as 2+2, then adding 8 + 2 = 10 and 10 + 2 = 12), involves mentally representing the magnitude of one addend as both a whole and as parts. Once children have access to multiple strategies, they typically select strategies adaptively, based on problem characteristics. When solving a problem that has one small addend, such as 8+3, a child might use count-on, but when solving a problem with two larger addends, such as 8+9, a child might use decomposition [25,26].

Children who persist in using count-all as their predominate strategy during early elementary school are more likely than those who shift to more advanced strategies to have lower mathematics achievement later in elementary school [16,27–30]. This trend is at least in part because count-all is increasingly inefficient and error prone as addition problems become more complex. As the size of addends increase, it becomes impossible to represent each addend and the sum with fingers and cumbersome to keep track while counting a large number of items. Thus, the use of more advanced strategies becomes critical for accurately solving problems with sums greater than ten [10].

Much of early instruction involves using objects and drawings to represent the quantities of addends to arrive at the sum [18]. Children use counting strategies more frequently when a task includes discrete quantities than when one involves purely continuous quantity [31]. Encouraging children to count discrete objects to solve addition problems may initially help them acquire a count-all strategy. If materials are consistently presented in this way, however, it may stymie their progression toward more advanced strategies and mentally representing addends. On the other hand, an approach that evokes a mental representation of the addends’ magnitudes may promote greater use of advanced strategies.

### Numerical magnitude knowledge

Children develop mental representations of single-digit number magnitudes gradually during early childhood. They first associate numerals with an absolute magnitude (3 equals * * *) [32,33]. Later, they refine these representations to include information about relative magnitude (5 is more than 3) such that they develop a mental number line (e.g., [29,30]). On this mental number line, numerical magnitude is represented spatially from smaller numbers on the left to larger ones on the right, with numbers distributed proportionally along the scale [34–37].

This knowledge is essential for arithmetic. It is both correlationally and causally related to arithmetic accuracy. By preschool, the accuracy of children’s judgments of the magnitude of Arabic numerals on a number line estimation task is related to their accuracy on addition problems [16,38]. Similarly, the speed and accuracy with which 6- and 8-year-olds determine which of two Arabic numerals is greater is related to their arithmetic fluency and predictive of later math achievement [39–41]. Interventions that improve the linearity of preschoolers’ and first graders’ estimates of numerical magnitude also improve their memory for the sums of arithmetic problems [42,43].

Numerical magnitude knowledge may contribute to arithmetic accuracy in at least three ways. First, a linear mental number line seems to constrain responses to addition problems. To judge whether a sum is reasonable, one must understand the relative magnitude of the addends and of the sum in relation to them (e.g., 8 + 4 cannot make 7 and cannot make 30). Indeed, children with more accurate and linear mental representations of relative numerical magnitude generate responses to arithmetic problems closer to the actual sum than those with poorer numerical magnitude knowledge [10,38,42–44].

Second, a mental number line involves spatial-numeric associations that seem to aid arithmetic operations [45,46]. Adults exhibit shifts in spatial attention to the right when performing addition, suggesting they internalize addition as movement toward larger numbers on a mental number line [47–49]. This effect also suggests that the spatial cues of magnitude play a role in generating and constraining responses to addition problems.

Finally, the ability to mentally represent the magnitude of numerals might support the use of more advanced strategies, which are associated with greater accuracy. There is already evidence that children who are more accurate at comparing Arabic numerals are more likely to use retrieval [15,41]. An idea explored in this study is that numerical magnitude knowledge might also aid the use of more advanced calculation strategies, such as count-on and decomposition. No previous work has examined whether interventions involving linear numerical magnitude understanding affect arithmetic calculation strategies in addition to memory for sums.

These findings raise the question: How can addition instruction be structured to capitalize on children’s mental representation of numerical magnitude? While various visual representations of quantity are used for math instruction in early childhood, many commonly used materials (e.g., pictures and objects arranged in arrays, dice with discrete canonical representations of dots) do not instantiate the linear and spatial features of a mental number line. These materials may not be as effective as possible because they do not activate children’s mental number line during arithmetic practice or help children discern the relevance of their mental representations of numerical magnitude for constraining their responses and executing better strategies.

### Present study

The purpose of the present study was to compare the effectiveness of instructional materials for addition practice. Our hypotheses were informed by the Cognitive Alignment Framework [11]. Because numerical magnitude knowledge is a mental representation essential to arithmetic accuracy, we hypothesized that materials aligned to its key linear-spatial features would promote greater learning, than those lacking this alignment. Specifically, we hypothesized that addition practice with materials that instantiate the linear and spatial features of a mental number line would activate children’s mental number line to promote greater use of advanced strategies and constrain children’s responses, thus leading to greater accuracy, than practice with materials that did not instantiate these mental number line features.

**Fig 1:**
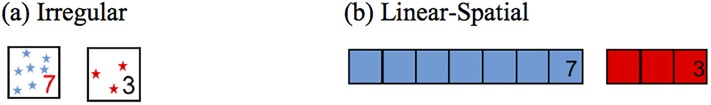
Examples of addend representations used in each condition: (a) irregular and (b) linear-spatial.

To test this hypothesis, low-income kindergartners were randomly assigned to practice addition problems with sums up to ten using one of two representations of the numerical magnitude of the addends 1–9: irregular or linear-spatial representations (see Fig 1). The irregular representation involved groups of discrete stars arranged in random arrays. The linear-spatial representation involved discrete squares organized linearly on horizontal sticks of varying length, such that the correlation between length and number of discrete squares provided a salient spatial-numeric association.

Our approach was novel relative to other studies that have examined the relation between linear numerical magnitude representations and arithmetic. Unlike Ramani and Siegler [42] for example, the objective of the training was not to improve children’s numerical magnitude knowledge and then examine whether there were spillover effects to their memory of addition sums. In fact, the training only included representations of the magnitudes of numbers up to ten, for which many kindergartners already have a linear representation [50]. Rather, the objective was to determine whether activating children’s existing mental representations using materials that instantiate its linear-spatial properties would help children discern the relevance of their numerical magnitude knowledge for addition and promote the use of more advanced mental addition strategies to solve unknown problems. Thus, we hypothesized that children who possessed greater initial numerical magnitude knowledge at the onset of instruction would be more likely to benefit from linear-spatial materials than those with lesser numerical magnitude knowledge as the materials would align with and activate their existing knowledge.

For this study, we chose specifically to focus on low-income children, given they are far more likely to use less sophisticated strategies [10]. Before and following practice, children were tested on novel arithmetic problems, involving sums larger than ten. We expected that children who practiced addition with the linear-spatial materials would demonstrate greater improvement in accuracy than children who practiced with irregular materials. We also expected this improvement would be mediated by a greater use of advanced strategies. Because the majority of assessment problems were different than those practiced and involved larger sums, improvement in accuracy could not be a result of improved memory of the sums.

In both practice conditions children were instructed how to use and assisted in using the count-on strategy to determine the sum. Despite practice with the strategy being equivalent for both groups, we expected that children who practiced with the linear-spatial materials would choose to use the strategy more frequently to solve unfamiliar addition problems following training than those who used the irregular ones. The logic was that because the linear-spatial materials aligned to the mental number line they would better enable children to activate their mental representations and, thus, implement and acquire the count-on strategy. In addition, we expected that increased use of advanced strategies would be most apparent among those children in the linear-spatial condition who possessed the greatest numerical magnitude knowledge before training. It was for those children that there would be the greatest alignment between the linear-spatial materials and their existing mental representations of numerical magnitude.

## Method

### Participants

This study included 29 kindergartners (mean age = 6 years, 3 months) from four classrooms in a low-income urban charter school. The percentage of children eligible for free or reduced lunch at the school was 84%. The school served primarily Hispanic and African American students. The *irregular condition* included 15 children and the *linear-spatial* condition included 14 children. A power analysis indicated that our sample size had 86% power to detect a medium effect size with an alpha of 0.05 for repeated measures ANOVAs. Genders were equally distributed across conditions (irregular: 7 males, 8 females; linear-spatial: 7 males, 7 females) and preliminary analyses showed no correlation between gender and variables of interest. Children were first matched by gender and then randomly assigned to conditions within classroom, such that children from each classroom were represented in each condition group.

Testing occurred in the spring of the school year. Testing of children in the two conditions was interleaved, so that children in them were matched for the time of year of their participation. By the time of testing, children had been exposed to arithmetic instruction. The common core standards, on which the curriculum was based, expect children to leave kindergarten able to solve addition and subtraction problems within 10 and to be able to find the number to make 10 when given any number between 1 and 9 [18].

Researchers received approval to conduct this research from their university’s Institutional Review Board. Additionally, a letter of approval was written by the principal of the participating school, permitting the researchers to recruit students. Both written parental consent and verbal child assent were obtained for each participant.

### Design

Children met individually with an experimenter for a total of 6 sessions over approximately 3 weeks. In sessions 1 and 6, children completed the pre- and posttests; in sessions 2–5, they played the Building Tens addition game. Children completed the posttest (session 6) on average one week after the final training (range was between 3 and 17 days). The study was carried out by four experimenters and each experimenter worked with children from both conditions.

#### Pretest/Posttest sessions (sessions 1 and 6)

In these sessions, children completed two tasks in a fixed order: Numeracy Screener then an Addition Accuracy and Strategy Assessment.

#### Numeracy Screener

The Numeracy Screener measures children’s accuracy comparing the numerical magnitudes of two Arabic numerals, and is an accepted measure of kindergartners’ numerical magnitude knowledge [51–54]. This task was previously shown to be highly reliable, *α* = .83 [54]. Children were presented with pairs of numerals between 1 and 9 and asked to cross out the larger of each pair. The order in which the magnitudes appeared was counterbalanced, such that the larger magnitude was on each side on half of the items. Children had two minutes to compare up to 56 pairs. A point was given for each pair on which the larger magnitude was correctly identified.

#### Addition Accuracy and Strategy Assessment

Children were presented 12 single-digit addition problems. The problems and orders in which they were presented are listed in S1 Table. The majority of the problems had not been practiced during training. Children saw the problems in a pre-determined order for pretest and in a different order for posttest. Children were presented one problem at a time, with the experimenter showing the problem (a + b) and saying it aloud (“How much is a + b?”), and were told to verbally provide the sum. Children were not provided any resources (e.g., paper and pencil, counters), but were allowed as much time as necessary to solve the problems. Children’s verbal responses were coded as correct or incorrect by the experimenter. Children’s absolute error on each problem was calculated as the absolute difference between the verbal sum they provided and the correct sum. Internal consistency of pretest scores was calculated using Cronbach’s alpha, and both absolute error (*α* = .90) and accuracy (*α* = .82) were determined to be reliable measures.

To determine the strategy used by participants, the experimenter recorded any overt signs of strategy use (e.g., if the child counted out-loud or used fingers) during problem solving. When there was not sufficient information to code the child’s strategy, the experimenter asked how he or she “figured it out.” This kind of combination of behavioral observations and retrospective self-reports has been found to lead to valid strategy classifications [55,56]. Sessions were audio-recorded, to allow researchers to confirm strategy approaches.

For each trial, children’s approach to solving the problems was classified as count-all, advanced strategy, or other. Children were coded as using a “count-all” strategy when they counted out each addend beginning with one. Children were coded as using an “advanced strategy” when they used one of the following strategies, which require mentally representing at least one addend: count-on, decomposition, or retrieval. Count-on involves counting up from one of the addends. Decomposition involves transforming the original problem into two or more simpler problems. Retrieval involves recalling the sum from memory and was only coded when children answered the problem within approximately three seconds. An “other” code was used when the child’s strategy did not align with one of the main strategy codes, when the child reported guessing, or when the child could not articulate or demonstrate a strategy. S2 Table provides further information about strategy coding. The data were coded independently by two raters, and their agreement rate was 91%. Raters consulted on all instances in which children’s reported strategy conflicted with their observed behavior and together agreed on the final code.

#### Game-playing sessions (Sessions 2–5)

In each of these sessions, children played the *Building Tens* game once with an experimenter during which they practiced combinations summing to ten. Children were randomly assigned to one of two experimental conditions that differed only in the representational format of the numerical magnitudes of the addends. In the *irregular condition*, the numerical magnitude of each addend was represented by discrete sets of stars organized in random arrays on one-inch square cards. In the *linear-spatial condition*, the numerical magnitude of each addend was represented as discrete units organized linearly using wooden sticks of varying lengths segmented into 2-cm. units. Fig 1 presents the addend representations used in each condition. The size of the discrete units used in the addend representations varied by condition, with the stars being smaller than the squares, because of their arrangement. However, the units in both conditions were of sufficient size to allow for enumeration.

The game boards for each condition on which children solved the problems were structured in the same way. Both game boards were 15” x 15” and included a visual representation of ten units at the top–ten stars in a random array or ten squares organized linearly. Below the representation of ten, both boards included a demarcated area for placing the addends for each of the nine practiced problems.

The game playing procedure was identical in both conditions. Each player had a set of pieces (blue for the experimenter and red for the child) representing the numerical values 1–9 both non-symbolically and with an Arabic numeral. During the game, the experimenter selected a numerical value and asked the child to find the numerical value that, when combined with the first, would equal ten. Once the child identified the correct addend and placed it on the board, the child was asked to find the symbolic equation that represented how they made ten (e.g., 5 + 5 = 10). If a child provided an incorrect response, the experimenter told the child whether the resulting sum was more or less than 10 (e.g., “oops! Look–this is more than 10!”) and asked the child to select a different numerical value. If the child was incorrect a second time, the experimenter told the child which addend to select. Finally, on each turn, the experimenter pointed to the pieces and the equation card and said, “Look! *[first number on equation card]* plus *[second number on equation card]* equals 10” (e.g., “Look! Five plus five equals 10”).

The sessions were designed to become increasingly challenging for the child, with the child taking on greater responsibility for using the count-on strategy between sessions 1 and 2 and practicing a greater variety of combinations from sessions 2 to 3 to 4 (see S3 Table for a list of pieces given to the experimenter and child on each session). The first time the game was played, all nine combinations of 10 that involved two addends were practiced (the five combinations and the associative pairs). At the end of each turn, the experimenter used the count-on strategy then verified the addition fact (e.g., “6…7, 8, 9, 10. 6 plus 4 equals 10”). The second time the game was played was identical to the first except that the child was expected to actively count-on. The experimenter said the value of the first addend. Then, the experimenter pointed to each unit in the second addend while the child counted-on. This procedure ensured that children in both conditions accurately enumerated the discrete units and did not miscount. The third time the game was played, children practiced the five combinations of ten, without commuted pairs (e.g., 9 + 1, but not 1 + 9 was practiced) during the first five turns. The last four turns they practiced combinations with three addends, in which the first addend was always 5. Children had the addends 1–4 and had to figure out a combination of five so that the overall sum would be ten. In this session, children were expected to count-on as they had done in the previous session, while the experimenter pointed to the units. The fourth time the game was played, children practiced combinations with three addends for seven out of the nine total turns. The first five turns involved re-practicing combinations of ten in which one of the addends was five. The children were given pieces representing 1 through 5, thus requiring children to select two addends for four out of the five trials. The last four turns involved practicing combinations in which the first addend was 4, three of which required children to use two addends to arrive at the value needed (i.e., 6) to arrive at a total sum of 10. To promote fluency and draw children’s attention to the whole magnitude, neither the child or the experimenter counted-on during the fourth session. In both conditions, each of the training sessions lasted about ten minutes and the experimenter provided the same assistance.

## Results

Preliminary analyses indicated random-assignment was successful: there were no differences between conditions in children’s age or their pretest performance on the numerical measures (numeracy screener, arithmetic absolute error, accuracy, and strategy categories). Table 1 presents children’s pretest mean score, by condition, and the *p*-values from independent sample *t*-tests comparing between conditions.

**Table 1:** Pretest mean scores for children in the irregular and linear-spatial conditions. Standard deviations are presented in parentheses. P-values were calculated from independent sample t-tests comparing children between the two training conditions.

	Irregular Materials	Linear-Spatial Materials	*p*-value
Age in months	75.59 (4.31)	73.42 (3.85)	.16
Numeracy Screener	34 (15.22)	40.50 (10.73)	.20
Arithmetic Percent Correct	24% (25)	33% (23)	.37
Arithmetic Absolute Error	3.04 (1.43)	2.53 (2.21)	.46
Use of Other Strategies	51% (40)	39% (39)	.42
Use of Count-All Strategy	35% (34)	42% (38)	.56
Use of Advanced Strategies^a^	14% (27)	19% (33)	.67

^a^Advanced strategies include count-on, decomposition, and retrieval

### Numerical magnitude knowledge

To test for condition effects on children’s numerical magnitude knowledge, we conducted a 2 (Condition: Irregular vs. Linear-Spatial) x 2 (Time: Pretest vs. Posttest) repeated measure ANVOA on the number of pairs for which children correctly identified the greater numeral. As expected, the results indicated no main effects or interactions. Children in neither condition demonstrated significant improvement between pretest and posttest (Irregular: *M* = 38.07, *SD* = 11.24; Linear-spatial: *M* = 41.93, *SD* = 9.47).

### Addition accuracy and strategies

We hypothesized the linear-spatial condition would lead to greater improvements in addition accuracy and strategies because it would align with children existing linear mental number lines. While the differences in numerical magnitude knowledge between conditions were not significant at pretest, to be as conservative as possible, we used children’s numeracy screener scores as a covariate in the following analyses testing condition effects on addition accuracy and strategies.

#### Accuracy

We chose to use absolute error as a measure of accuracy–in addition to using percentage or problems answered correctly–because absolute error is a more sensitive measure of improvement in addition than percentage correct. Children’s absolute error was calculated for each problem separately then averaged: AE = |correct answer–child’s response|. High absolute error indicates a response far from the actual sum and, thus, a more implausible response. In contrast, low absolute error indicates that while a response may be incorrect it is likely constrained by an understanding of numerical magnitudes or due to minor error executing an appropriate strategy (e.g., counting on one to few when using count-on).

**Fig 2:**
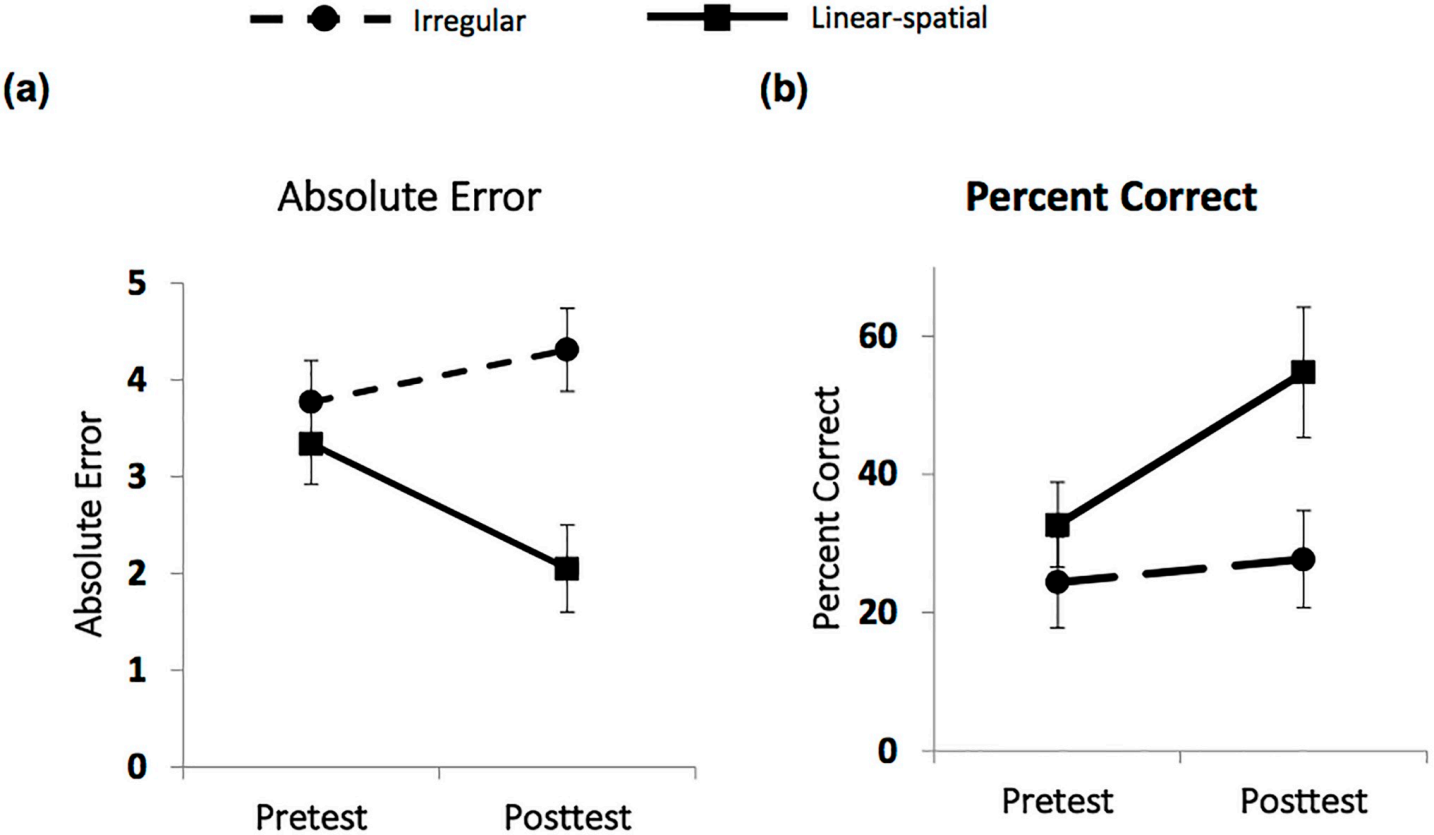
Pretest to posttest improvement in (a) absolute error and (b) percent correct on addition problems for children who practiced with irregular versus linear-spatial materials.

A repeated measures ACNOVA on children’s average absolute error, controlling for their numeracy screener score, found a condition by time interaction, *F*(1,26) = 6.63, *p* = .01, *η*_*p*_^*2*^ = .20. As shown in Fig 2a, the mean absolute error of children in the linear-spatial condition decreased from 2.53 (*SD* = 2.20) at pretest to 1.51 (*SD* = 1.77) at posttest, *p* = .01. In contrast, children in the irregular condition exhibited no improvement in absolute error (from *M* = 3.04, *SD* = 1.43 at pretest to *M* = 3.26, *SD* = 1.60 at posttest, *p* = .47. In other words, the absolute error of children in the linear-spatial group decreased by about half a standard deviation from pretest to posttest, (Hedges’ *g* = -.50), while the absolute error of children in the irregular condition essentially did not change (increased by only about one-tenth of a standard deviation, Hedges’ *g* = .14).

It was possible that this finding was due to a greater number of correct trials in the linear-spatial condition. To test this possibility, we calculated absolute error using only incorrect items. A similar pattern of results was found: the condition by time interaction effect remained, *F*(1,26) = 10.67, *p* = .003, *η*_*p*_^*2*^ = .29, and was explained by a decrease in implausible responses in the linear-spatial condition (from *M* = 3.7, *SD* = 1.91 at pretest to *M* = 2.09, *SD* = 1.67 at posttest; *p* = .01, Hedges’ *g* = -.87, but not in the irregular condition (from *M* = 3.36, *SD* = 1.16 at pretest to *M* = 4.08, *SD* = 1.39 at posttest, *p* = .07, Hedges’ *g* = .47).

An examination of the percentage of problems for which children provided the correct sum showed parallel results. A 2 (Condition: Irregular vs. Linear-Spatial) x 2 (Time: Pretest vs. Posttest) ANCOVA on the percentage of addition problems children solved correctly found a time by condition interaction, *F*(1,26) = 4.615, *p* = .05, *η*_*p*_^*2*^ = .14. Although children received no practice with the addition problems used in the assessment, the mean percent of problems children in the linear-spatial condition solved correctly increased substantially, from 33% (*SD* = 23) at pretest to 55% (*SD* = 34) at posttest, *p* = .002, Hedges’ *g* = .74. In contrast, children in the irregular condition did not exhibit improvement in the percent of problems solved correctly from pretest (*M* = 24%, *SD* = 25) to posttest *(M* = 28%, *SD* = 27), *p* = .52, Hedges’ *g* = .15, (Fig 2b).

#### Strategies

Table 2 presents the frequency with which children used each strategy on the arithmetic assessment at pretest and posttest by condition. Separate 2 (Condition: Irregular vs. Linear-Spatial) x 2 (Time: Pretest vs. Posttest) repeated measure ANCOVAs were conducted on the percentage of problems, controlling for numeracy screener, children used each strategy category: (1) an ineffective strategy (“other” code), (2) count-all, and (3) an advanced mental strategy (count-on, decomposition, and retrieval). The percentage of problems on which children used an “other” strategy and a count-all strategy did not change from pretest to posttest across or within either condition. For use of advanced strategies, however, there was a time by condition interaction, *F*(1,26) = 5.87, *p* = .02, *η*_*p*_^*2*^ = .18. Children in the linear-spatial condition more than doubled the number of problems on which they used an advanced strategy, from 19% of problems (*SD* = 33) at pretest to 52% of problems at posttest (*SD* = 45), *p* < .001, Hedges’ *g* = .81. In contrast, children in the irregular condition did not show any change in the frequency with which they used advanced strategies (Pretest: 14% (*SD* = 27); Posttest: 19% (*SD* = 28), *p* = .46, Hedges’ *g* = .18).

**Table 2:** Pretest to posttest change in strategy choice for children in the irregular and linear-spatial conditions. Shown is the percent of problems (SD) on which each strategy was used.

	Irregular Materials		Linear-Spatial Materials	
	Pretest	Posttest	Pretest	Posttest
Other	51 (40)	49 (39)	39 (39)	22 (36)
Count All	35 (34)	32 (34)	42 (38)	36 (33)
Advanced Strategies^a^	14 (27)	19 (28)	19 (33)	52 (45)
Count-On	11 (23)	13 (17)	16 (32)	44 (42)
Decomposition	0 (0)	0 (0)	1 (2)	1 (3)
Retrieval	4 (8)	6 (13)	2 (6)	7 (9)

^a^Composite of all three advanced strategies

The linear-spatial condition not only increased the frequency with which children attempted advanced strategies, but also improved how accurately they executed them. A repeated measures ACNOVA on the number of problems, out of 12 total, for which children used an advanced strategy to reach a correct sum revealed a condition by time interaction, *F*(1,26) = 5.37, *p* = .03, *η*_*p*_^*2*^ = .17. Children in the linear-spatial condition correctly used advanced strategies on a greater number of problems at posttest than pretest, 4.50 (*SD* = 4.47) vs. 1.50 (*SD* = 2.38), respectively, *p* < .001, Hedges’ *g* = .81. In contrast, children in the irregular condition did not show higher accurate use of advanced strategies at posttest verses pretest, 1.67 (*SD* = 2.99) vs. 1.07 (*SD* = 2.55), *p* = .26, Hedges’ *g* = .81. There were no pretest to posttest changes or time by condition interactions in the accuracy in which children used other or count-all strategies for children in either condition.

Finally, to gain a deeper understanding of children’s use of advanced strategies, we conducted a MANCOVA testing for differences in the proportion of problems on which children attempted to use the three advanced strategies: count-on, decomposition, and retrieval. For the count-on strategy, the results indicated a time by condition interaction, *F*(1,26) = 5.48, *p* = .03, *η*_*p*_^*2*^ = .17. There were no condition differences at pretest, but at posttest, children in the linear-spatial condition attempted count-on more frequently than children in the irregular condition, *p* = .04, Hedges’ *g* = .95. There were no effects for use of decomposition or retrieval.

Similarly, a MANCOVA examining changes in children’s accuracy of executing the advanced strategies found only a time by condition interaction for count-on: *F*(1,26) = 6.24, *p* = .02, *η*_*p*_^*2*^ = .19. Children in the linear-spatial condition accurately executed count-on on a greater number of problems than children in the irregular condition at posttest, 3.79 (*SD* = 4.06) vs. 1.07 (*SD* = 1.67), respectively, *p* = .056, Hedges’ *g* = .86.

### Mediation analysis: Condition influence on accuracy via strategy

To examine whether improvement in accuracy in the linear-spatial condition was mediated by the increased use of advanced strategies in that condition, we conducted a mediation analysis. We tested a model in which the percentage of problems on which children used advanced strategies at posttest served as a mediator of the association between pretest condition (predictor) and posttest absolute error (outcome), with pretest absolute error as a covariate on the outcome. As noted earlier, we chose to examine absolute error as the outcome, rather than percentage correct, because using advanced strategies should lead both to a greater number of correct responses and responses closer to the actual sum even if minor errors in execution lead to an incorrect response.

The mediation analysis was conducted using the SPSS macro PROCESS, which estimates confidence intervals using bias-correcting bootstrapping [57]. Bootstrapping is advantageous, particularly with small sample sizes, because it does not assume normal distribution of the indirect effect. The analysis used 10,000 bootstrap samples with replacement from the sample to calculate point estimates. The 95% confidence interval was then determined based on sampling distribution of the point estimates [58]. The indirect effect is considered statistically significant if the 95% confidence interval does not include zero [57].

**Fig 3:**
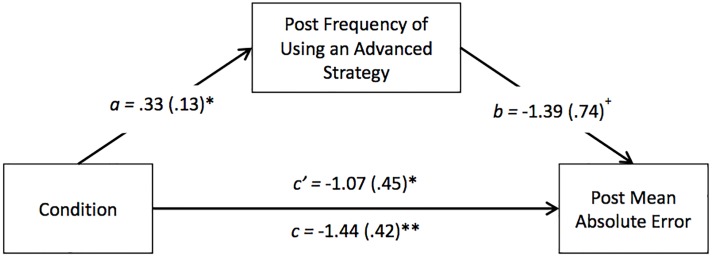
Improvement in advanced strategies as a mediator of the relation between condition and addition absolute error at posttest. Pretest absolute error was used as a covariate on the outcome. ^+^*p* < .10, *p < .05, **p < .01.

Fig 3 presents the results of the mediation analysis. The direct effect of training condition on addition absolute error (*b* = -1.07; *p* = .03; confidence interval (CI): [-1.99, -.15] was substantially lower than when the relation was examined without controlling for the percentage of problems on which children used an advanced strategy (*b* = -1.44, B = -.41, *p* = .002). The product of the coefficients (ab) for the indirect path from training condition to absolute error by way of frequency of using an advanced strategy was significant (point estimate = -.46; 95% bias-corrected; confidence interval (CI): [-1.47 to -.05], indicating that use of advanced strategies mediated the relation between training condition and improvement in absolute error.

### Numerical magnitude knowledge as a predictor of advanced strategies

**Fig 4:**
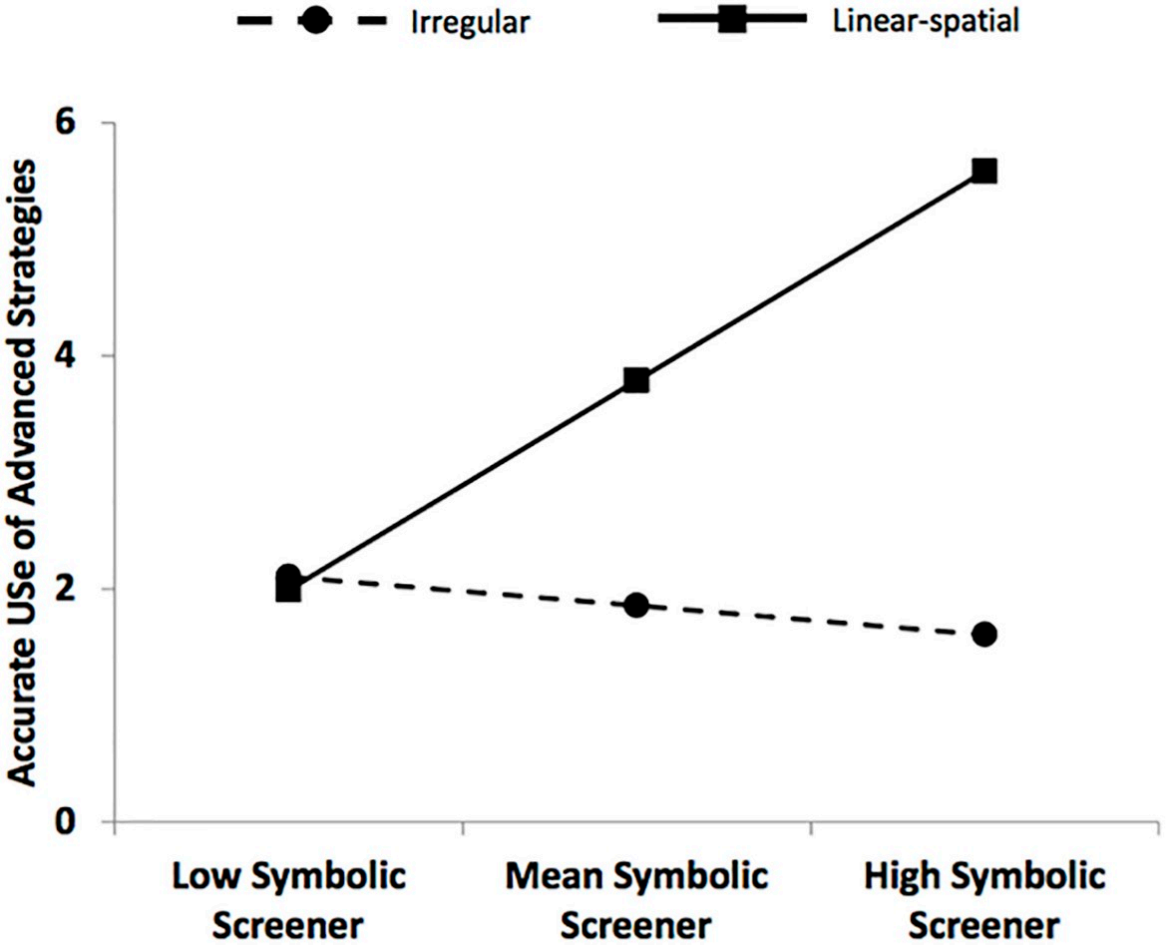
Conditional effect of pretest numerical magnitude knowledge on the number of problems accurately answered via an advanced strategy on the arithmetic assessment at posttest. Predicted accuracy scores were calculated at -1, 0, and 1 SD from the mean on the Numeracy Screener.

Finally, we tested the prediction that the linear-spatial materials would be most effective in promoting advanced strategy execution for children with greater numerical magnitude knowledge at the onset of instruction. We conducted a multiple regression with pretest Numeracy Screener, condition (0 = irregular, 1 = linear-spatial), and a pretest Numeracy Screener by condition interaction as predictors of the number of problems for which children accurately used advanced strategies. After partialing out the variance for pretest accurate use of advanced strategies, the Numeracy Screener by condition interaction was significant, *b* = .15, *SE* = .07, *β* = .29, *t* = 2.13, *p* = .04. A positive relation was evident between the number of pairs on which children had correctly identified the greater numeral at pretest and accurate use of advanced strategies at posttest for children who had practiced with the linear-spatial materials (*p* = .03). However, as shown in Fig 4, this association was not apparent for children who had practiced with the irregular materials.

## Discussion

The present findings demonstrate that the benefits of addition practice using linear-spatial materials are greater than benefits of practice using materials with irregular groups of discrete pictures. The linear-spatial materials instantiated the key properties of the mental number line believed to contribute to arithmetic accuracy and fluency, whereas, the irregular materials did not. Low-income kindergartners who practiced addition sums up to ten using linear-spatial materials to represent the magnitudes of the addends showed marked improvement in their solutions to novel addition problems, whereas those who practiced with irregular arrays did not. Even when they answered incorrectly, children in the linear-spatial condition were more likely to provide responses closer to the actual sum, than those in the irregular condition. Further, children who practiced addition with the linear-spatial materials used advanced strategies, particularly count-on, with greater frequency and accuracy than those who learned with the irregular materials. This difference in strategy choice mediated the association between training materials and arithmetic accuracy. These results converge with those of previous research demonstrating the importance of alignment for learning and memory encoding [11,59]. The implications of these findings for advancing instructional design theory as well as early arithmetic instruction are discussed below.

### The cognitive alignment framework

The cognitive alignment framework offers a theoretical view of instructional design. It posits that the more precisely physical materials and learning activities align with the desired mental representation, the more likely students are to acquire that representation. Key features of the underlying mental representations must be instantiated in the physical materials for learning of that representation to occur. This approach has successfully predicted which numerical board games [11], patterning activities [60], and games for extending the count sequence [61] would be most effective.

The present findings provide further evidence for the cognitive alignment framework by extending its application to arithmetic. The increased use of a count-on strategy among children in the linear-spatial materials condition, but not among those in the irregular materials condition, is most noteworthy. Previous research has shown that improving children’s numerical magnitude knowledge using linear number board games can, in turn, improve their memory of simple addition sums [42]. This was the first study to demonstrate that linear-spatial materials can affect children’s procedural strategies for solving addition problems.

Further, unlike in previous studies, this finding cannot be attributed to differential improvement in numerical magnitude knowledge. In both conditions, children’s accuracy comparing symbolic magnitudes did not change from pretest to posttest. Rather, it was children’s existing knowledge of numerical magnitudes prior to training that moderated improvement. For those children who were trained with the linear-spatial materials, initial accuracy when comparing symbolic numeric magnitudes predicted the frequency with which they used advanced strategies at posttest, even after controlling for pretest strategy usage.

The increased use of count-on in the linear-spatial condition also cannot be attributed to differences in practice. Children in both conditions were provided equivalent practice using the count-on strategy during training. Practice of the strategy, in absence of an alignment (i.e., children with lower numerical magnitude knowledge in the linear-spatial condition or irregular materials) was not sufficient to promote its use. Thus, the pattern of results supported the hypothesis that the alignment between the linear-spatial materials and children’s existing linear mental representations would activate children’s existing linear mental representations and highlight the relevance of this knowledge for executing arithmetic strategies and constraining responses.

Besides extending the application of cognitive alignment to a new aspect of mathematics, the present findings also demonstrate that it is useful in situations where there is an indirect mapping via an intermediary representation underlying a desired procedural outcome (e.g., linear-spatial materials to linear magnitude representations to arithmetic strategies). Earlier studies of the efficacy of the cognitive alignment framework have involved a direct mapping between the materials and the desired outcome. For example, past research tested the extent to which a linear number board game improves linear representations of numerical magnitude [11,44]. In contrast, this study tested the hypothesis that greater learning would occur if instructional materials instantiated the key properties of a mental representation that is critical for, but not the same as, the desired outcome. Both sets of materials provided a representation of addition as combining parts to form a whole–the direct outcome measure of the study. However, only the linear-spatial materials instantiated the mental number line believed to be involved in arithmetic. Thus, finding that the linear-spatial materials promoted greater improvement, particularly for children who demonstrated better numerical knowledge prior to instruction, could be attributed to the activation of this relevant prior knowledge.

This contribution is important given the wide range of research that demonstrates the importance of activating relevant prior knowledge for strategy choice and conceptual change [62–64]. Suggestions for activating students’ prior knowledge often involve probing questions (e.g., what does this remind you of? Do you know anything that can help?) or having students generate their own solutions before receiving explicit direction (e.g., [65–67]). These approaches may have limited utility with younger children who are less able to reason about or articulate their thinking [68]. In the case of this study, cognitive alignment of the physical materials activated relevant prior knowledge—the mental number line—without the need for verbal explication.

### Implications and future directions

These findings also have practical implications for classroom instruction. They provide evidence that representing quantities linearly, rather than as random collections of objects or drawings, would be most efficacious for arithmetic instruction in early elementary school. There are various linear materials already available in classrooms that could be utilized and whose efficacy might be tested. These include Unifix Cubes, where cubes are attached to represent larger quantities, and base-ten materials.

In addition to activating the linear mental representations of numerical magnitude, the linear-spatial materials might have been effective because they supported the comparison of magnitudes. Comparison is a powerful learning mechanism that allows children to extract the relevant properties of objects [13,69]. The correlation between length and magnitude was more salient with the linear-spatial materials than that between surface area and magnitude in the irregular materials. This difference between materials may have allowed children to more easily compare the magnitudes of the addends. This comparison process may have promoted an understanding of relative magnitudes and of which sums could plausibly result from combining two addends. Explicitly directing children to compare addends may strengthen the value of linear-spatial materials.

The current findings also suggest that a deliberate focus on helping children learn the relative magnitude of numerals before and while teaching arithmetic could maximize the benefits of arithmetic practice. Previous correlational work has documented a relation between linear representations of number and the use of more advanced strategies [70,71]. In this experimental study, children in the linear-spatial materials condition who had the greatest numerical magnitude knowledge before training demonstrated the greatest increase in the use of count-on. Thus, these findings and previous ones suggest that arithmetic instruction might be the most effective for children who already have an adept representation of symbolic numerical magnitude.

In considering these possible implications, limitations of this study should be noted. The current study included a limited number of children and examined short-term improvement. Future research should test these materials with a larger and more diverse group of students as well as examine whether the benefits persist over time. Further, while the measures used in the study (solutions provided for arithmetic problems) are unlikely to be susceptible to bias, a blinded replication of the study may be worthwhile.

In conclusion, the present study provides evidence that practicing arithmetic and a count-on strategy with linear-spatial materials leads to improvement on unpracticed arithmetic problems, and higher spontaneous usage of advanced strategies. Children appeared to learn how to generalize knowledge they gain about relative magnitudes and solving addition problems beyond the training task. The effect sizes for the differences in absolute error and strategy choice were medium to high [72], and were in the range of other effect sizes from similar small-scale experiments (e.g., [73,74]). The findings support the importance of carefully designing and selecting materials for instruction in order to maximize learning, and the applicability of developmental psychology for instructional design.

## Supporting information

S1 Table The order of addition problems presented at pretest and posttest. (DOCX)

S2 Table Strategy coding with definitions and examples for how to solve the problem 3 + 8. (DOCX)

S3 Table List of each player’s addends during each game training session. (DOCX)

S1 Dataset (XLSX)
