# Supplementary material for: Materials count: Linear-spatial materials improve young children’s addition strategies and accuracy, irregular arrays don’t
Source: PLoS One. 2018 Dec 31;13(12):e0208832. doi: 10.1371/journal.pone.0208832 (PMC6312299; doi:10.1371/journal.pone.0208832)
Supplement: S1 Table — (DOCX) [file pone.0208832.s001.docx]

**S1 Table**. **The order of addition problems presented at pretest and posttest.**

| Pretest Order | Posttest Order |
| --- | --- |
| 2 + 6 | 5 + 4 |
| 5 + 4 | 4 + 2 |
| 3 + 8 | 5 + 9 |
| 7 + 5 | 9 + 8 |
| 3 + 4 | 2 + 6 |
| 6 + 3 | 3 + 7 |
| 4 + 7 | 7 + 5 |
| 6 + 5 | 3 + 8 |
| 4 + 2 | 3 + 4 |
| 5 + 9 | 6 + 5 |
| 9 + 8 | 4 + 7 |
| 3 + 7 | 6 + 3 |
