# Supplementary material for: Materials count: Linear-spatial materials improve young children’s addition strategies and accuracy, irregular arrays don’t
Source: PLoS One. 2018 Dec 31;13(12):e0208832. doi: 10.1371/journal.pone.0208832 (PMC6312299; doi:10.1371/journal.pone.0208832)
Supplement: S2 Table — (DOCX) [file pone.0208832.s002.docx]

**S2 Table. Strategy Coding with definitions and examples for how to solve the problem 3 + 8.**

| **Strategy** | **Definition** | **Example** | **Notes** |
| --- | --- | --- | --- |
| Count-All | Count out full sum beginning at 1 | count on fingers and mouthed “1-3”, “1-8”, and then “1-11” | Also coded as count-all if child just counted 1-11 without first counting each addend separately |
| Count-On | Count on from one addend | count out loud “9, 10, 11” | Could count from smaller addend (e.g., counting 4-11) or larger addend (e.g., counting 9-11) |
| Decomposition | Break a problem into simpler problems | “I know 2 plus 8 equals 10 and I added 1 more so it is 11” | Could decompose problems in various ways, including relying on sums to 10 or other memorized facts, such as twins (e.g., solving 4+5 by first recalling 4+4=8 and then 9+1=9) |
| Retrieval | Recall answer from memory | Responded immediately with “11.” When probed, “I just knew it” | Child quickly stated answer (within 3 seconds). Audio recordings were available to confirm latency. |
| Other | Strategy that cannot be coded into other codes | “83 because 8+3=83” OR “I just guessed that 8+3=20.” | Common examples of “other” were guessing, concatenating digits, stating one more than an addend, or stating one of the addends as the sum |
