# Supplementary material for: Materials count: Linear-spatial materials improve young children’s addition strategies and accuracy, irregular arrays don’t
Source: PLoS One. 2018 Dec 31;13(12):e0208832. doi: 10.1371/journal.pone.0208832 (PMC6312299; doi:10.1371/journal.pone.0208832)
Supplement: S3 Table — (DOCX) [file pone.0208832.s003.docx]

**S3 Table. List of each player’s addends during each game training session**

| **Training Session** | **Adult’s Addends** | **Child’s Addends** |
| --- | --- | --- |
| Session 1 | 1, 2, 3, 4, 5, 6, 7, 8, 9 | 1, 2, 3, 4, 5, 6, 7, 8, 9 |
| Session 2 | 1, 2, 3, 4, 5, 6, 7, 8, 9 | 1, 2, 3, 4, 5, 6, 7, 8, 9 |
| Session 3 | 1, 3, 4, 5, 5, 5, 5, 5, 8 | 1, 1, 2, 2, 2, 3, 3, 4, 4, 5, 6, 7, 9 |
| Session 4 | 5, 5, 5, 5, 5; 4, 4, 4, 4 | 1, 1, 2, 2, 3, 3, 4, 4, 5; 1, 2, 3, 3, 4, 5, 6 |
